# A Simplified Wheat Protoplast Transformation System and Guideline for Avoiding Protein Localization Artifacts

**DOI:** 10.3390/plants15111707

**Published:** 2026-05-31

**Authors:** Leyan Li, Shuai Zhong, Shuai Liu, Fan Zhang, Zehui Liu, Ruofei Wang, Yue Zhao, Qianwen Liu

**Affiliations:** 1 State Key Laboratory of High-Efficiency Production of Wheat-Maize Double Cropping, College of Life Science, Henan Agricultural University, Zhengzhou 450046, China; 2 State Key Laboratory of Cotton Bio-Breeding and Integrated Utilization, Institute of Cotton Research, Chinese Academy of Agricultural Sciences, Anyang 455000, China

**Keywords:** *Triticum aestivum* L., DNA transformation, protoplast, transient expression, fluorescent protein, subcellular localization, phase separation

## Abstract

The transient protoplast transformation system is a vital tool for studying protein subcellular localization and phase separation in wheat. However, current protocols remain underdeveloped, and the lack of systematic vector design analysis frequently leads to localization artifacts. Here, we established a simplified wheat mesophyll protoplast transformation method featuring a shortened cycle, streamlined handling, and no variety limitations, enabling stable acquisition of high-quality confocal imaging data. Using this method, we systematically examined the effects of the fluorescent tag position (N- vs. C-terminal) and promoter type (native, single *CaMV35S* and double *CaMV35S*) on protein localization and phase separation. Tag position proved decisive: improper fusion can affect the recognition of localization signals, leading to inaccurate patterns. Regarding promoters, the native promoter represents the optimal choice for physiological accuracy. Constitutive strong promoters such as *CaMV35S* boost gene expression and thereby enhance fluorescent signals for easier imaging, but overexpression may compromise localization fidelity and exacerbate molecular crowding effects, resulting in false-positive phase-separated aggregates. Conversely, insufficient expression will lead to false-negative outcomes. This standardized transformation system and the defined vector design principles offer a robust framework for minimizing artifacts in wheat protein localization and phase separation research.

## 1. Introduction

As one of the world’s most important food crops, the yield stability and quality improvement of wheat are directly related to global food security [1]. In-depth analysis of the molecular mechanisms underlying key biological processes such as wheat growth, development, and stress adaptation can reveal the expression patterns of critical genes at the fundamental theoretical level, providing important targets for breeding practices aimed at high yield and stress resistance. Protein subcellular localization is critical for elucidating molecular mechanisms [2,3].

Common methods for subcellular localization include subcellular fractionation, immunofluorescence, immunoelectron microscopy, and various live-cell imaging approaches [4]. Subcellular fractionation provides component-enriched protein abundance data but lacks single-cell resolution and is prone to cross-contamination [5]. Immunofluorescence and immunoelectron microscopy detect endogenous proteins with high precision, yet both rely on specific antibodies and require fixation and permeabilization steps that may disrupt fine cellular structures [6], and are unsuitable for dynamic observation in living cells [7]. Among live-cell imaging strategies, agroinfiltration is efficient in dicots yet limited in wheat [8]; stable transgenic lines provide the most physiological context but are time-consuming to generate [9]; and particle bombardment allows direct tissue transformation but often causes mechanical damage and overexpression artifacts [10]. Furthermore, heterologous expression in model plants such as Nicotiana benthamiana may also generate artifacts due to differences in the intracellular environment [11]. Therefore, the wheat protoplast transformation method accommodates the requirements for protein localization to be examined in a physiological context, to be visualized, and to be high-throughput and low-cost. It balances the experimental duration with the data quality, making it the preferred approach for observation of subcellular localization of wheat proteins.

Protoplast-based gene editing methods in wheat have been initially established [12] and continuously optimized [13,14,15,16,17]. The existing transformation systems are primarily oriented toward improving gene editing efficiency; by optimizing delivery methods, editing tools, and expression systems, they enable rapid validation of editing outcomes such as gene knockout and base substitution at the wheat cellular level. Notably, the optimization trajectory of these established transformation systems has consistently focused on how to obtain gene editing events more efficiently, rather than prioritizing the specific requirements of subcellular localization observations—such as the integrity of cellular physiological states and control of background signals. In consequence, we urgently require a protoplast transformation method that is specifically suitable for microscopic observation of the subcellular localization of target proteins.

Among the numerous molecular mechanisms dependent on subcellular localization, liquid–liquid phase separation has garnered significant attention in recent years [18,19,20]. As an important molecular assembly mechanism, liquid–liquid phase separation can drive proteins to form dynamic, regulatable membraneless organelles, enabling efficient enrichment and isolation of signaling molecules, thereby allowing cells to respond rapidly to environmental changes [21]. This mechanism has been confirmed to be widely involved in processes such as plant growth and development, signal transduction, and stress responses [22,23], and also plays key roles in various biological functions in wheat. For example, the wheat nuclear protein TaPSTE forms biomolecular condensates via phase separation to recruit the transcription factor TaNF-YC, activating ROS burst and Ca^2+^ influx to mediate stripe rust resistance [24]; the Rht8-encoded RNHL1 protein forms nuclear condensates via phase separation, interacting with TaEIL1 to form transcriptional hubs that regulate ethylene and gibberellin signaling to control plant height [25]. Notably, the occurrence and regulation of phase separation are highly dependent on the subcellular localization of proteins—the unique microenvironment of different cellular compartments directly affects the protein aggregation status and condensate formation capability [26,27,28,29].

Concurrently, we noted that certain common oversights in previous live-cell protoplast localization studies can compromise data interpretation: inappropriate tag selection can cause localization interference [30,31], and improper promoter choice can lead to overexpression aggregation [32]. Such artifacts are particularly problematic for phase separation studies, obscuring genuine phenomena. Accordingly, we conducted parallel comparisons of the effects of different tag positions (N-terminal vs. C-terminal fusion tags) and different promoters (native promoter, single *CaMV35S* and double *CaMV35S*) on the expression and behavior of target proteins.

In summary, this study establishes a simplified protoplast transformation system and put forward a guideline—including tag position and promoter selection—to minimize non-physiological artifacts.

## 2. Results

### 2.1. Efficiency and Reproducibility of the Standardized Procedure

**Figure 1:**
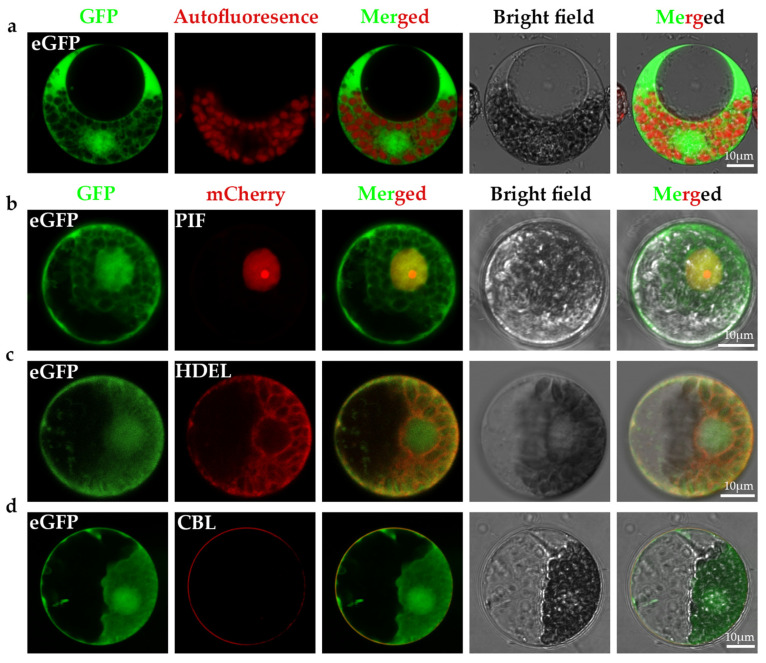
Localization of free GFP and its co-localization with nuclear, ER and plasma membrane markers in wheat protoplasts. (**a**) pCambia1305-eGFP (empty vector control). (**b**) pCambia1305-eGFP + pCambia1300-PIF-mCherry (nuclear marker). (**c**) pCambia1305-eGFP + pCambia1300-HDEL-mCherry (ER marker). (**d**) pCambia1305-eGFP + pCambia1300-CBL-mCherry (plasma membrane marker). Images were captured using confocal laser scanning microscopy (CLSM). Scale bars = 10 μm. GFP: excited at 488 nm, emission detected at 500–565 nm (green). Autofluorescence: excited at 488 nm, emission detected at 560–600 nm (red). mCherry: excited at 587 nm, emission detected at 610 nm (red).

The wheat mesophyll protoplast isolation and transformation method presented here is not influenced by variety and yields high-quality protoplasts. By using etiolated seedlings to avoid autofluorescence interference, omitting the plasmolysis step, reducing the enzyme concentration while cutting the digestion time by half, and relaxing the strict requirement for cell concentration during resuspension, these simplifications maintain the protoplast yield and viability while substantially shortening the experimental cycle. The parallel transformation and confocal imaging workflow established in this study operates stably, yielding high-quality visualization data. Free eGFP localization is shown in the figure, and the nuclear [33,34], cytoplasmic [35], and membrane [36] markers co-transformed with it also exhibit correct localization (Figure 1).

### 2.2. Effects of Fluorescent Tag Fusion Position and Promoter Type on Protein Localization

**Figure 2:**
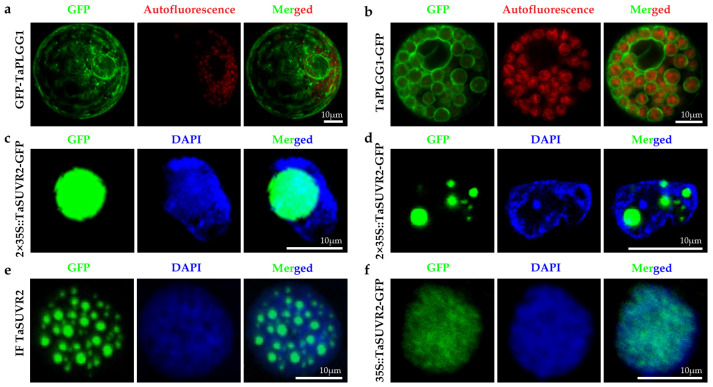
Comparison of different fluorescent tag positions and different promoter selections. (**a**) Wheat protoplasts transformed with pJIT163-2 × *CaMV35S*-eGFP-TaPLGG1. (**b**) Wheat protoplasts transformed with pCambia1305-2 × *CaMV35S*-TaPLGG1-eGFP. (**c**,**d**) Wheat protoplasts transformed with pCambia1305-2 × *CaMV35S*-TaSUVR2-eGFP; the nucleus is indicated in the figure. (**e**) Immunofluorescence detection of endogenous TaSUVR2 in wheat protoplast; the nucleus is indicated in the figure. (**f**) Wheat protoplasts transformed with pCambia1305-*CaMV35S*-TaSUVR2-eGFP; the nucleus is indicated in the figure. Images were captured using confocal laser scanning microscopy (CLSM). Scale bars = 10 μm. GFP: excited at 488 nm, emission detected at 500–565 nm (green). Autofluorescence: excited at 488 nm, emission detected at 560–600 nm (red). DAPI: excited at 405 nm, emission detected at 430–470 nm (blue).

We selected the wheat chloroplast protein PLGG1 as an auxiliary subject and systematically analyzed the effect of fluorescent tag fusion position (N-terminal vs. C-terminal) on protein subcellular localization. Sequence prediction indicated that this protein is chloroplast-localized, with its localization signal residing within amino acids 1–96 at the N-terminus. We examined the localization patterns under different tag configurations. When GFP was fused to the N-terminus of PLGG1, the fluorescence signal appeared in the cytoplasm rather than the chloroplast (Figure 2a). When GFP was fused to the C-terminus, the fluorescence signal overlapped with chloroplast autofluorescence (Figure 2b). Thus, the location of GFP tag fusion does indeed affect protein localization.

To verify the influence of different promoters on protein localization, we employed TaSUVR2 as an auxiliary subject. TaSUVR2 is a nuclear-localized wheat histone methyltransferase with punctate aggregation properties. We isolated protoplasts from wheat leaves and performed immunofluorescence detection of endogenous TaSUVR2. The endogenous protein appeared as fine puncta within the nucleus. We further examined the localization patterns driven by different heterologous promoters (Figure 2e). Under the double *CaMV35S* promoter, two patterns were observed: in some cells, the GFP signal formed a single large spherical body within the nucleus, while in others the signal appeared as fine puncta (Figure 2c,d). Under the single *CaMV35S* promoter, the GFP signal filled the nucleus uniformly, with no discernible puncta or bodies (Figure 2f). Thus, the type of promoter does indeed affect the protein punctate aggregation behavior.

## 3. Discussion

The wheat mesophyll protoplast transient expression system offers a balance of near-physiological relevance, direct visualization, high-throughput, and low-cost, making it the preferred choice for subcellular localization observation.

In previous protocols, protoplast isolation from wheat typically used green leaf tissue, included a plasmolysis step before enzymatic digestion, employed higher enzyme concentrations with longer digestion times (4–5 h), maintained a strict requirement for cell concentration during resuspension, and was primarily designed for genome editing efficiency evaluation [14]. Here, we made targeted adjustments: we used etiolated seedlings to avoid chlorophyll autofluorescence interference, omitted the plasmolysis step to avoid a non-physiological microenvironment, reduced the enzyme concentration and shortened the enzymatic hydrolysis time to 2–3 h to maintain milder digestion conditions, relaxed the strict requirement for cell concentration during resuspension to increase the experimental tolerance, and tailored the system for subcellular localization and punctate aggregation observation. These simplifications ensured sufficient protoplast quality while substantially shortening the experimental cycle, making the protocol more suitable for live-cell confocal imaging with reduced artifacts. Our system is suitable for stable readouts such as nuclear localization. For readouts involving the secretory/endomembrane system, or those highly dependent on physiological status, we explicitly recommend cross-validation on a case-by-case basis [37,38].

Regarding vector design, our systematic evaluation revealed that both tag position and promoter selection critically influence localization accuracy and phase separation readouts. For tag position, using the chloroplast protein PLGG1 as a test case, N-terminal GFP fusion resulted in cytoplasmic retention, whereas C-terminal fusion produced the expected chloroplast localization. Sequence analysis indicated that the PLGG1 localization signal resides within the N-terminal 1–96 amino acid region; placement of GFP at the N-terminus likely obstructs recognition of this signal, preventing correct targeting. We therefore recommend sequence analysis and structural prediction of the target protein to guide tag placement, with parallel N-terminal and C-terminal fusion experiments to determine the optimal configuration. For promoter selection, immunofluorescence detection of endogenous TaSUVR2 revealed fine punctate condensates uniformly distributed within the nucleus, representing the native punctate aggregation state (Figure 2e), and free GFP showed a diffuse localization pattern (Figure 1). In contrast, the double *CaMV35S* promoter produced irregular punctate structures—clear false-positive artifacts—while the single *CaMV35S* promoter failed to support formation of authentic condensates, resulting in a diffuse nuclear signal devoid of puncta, a false-negative outcome (Figure 2c,d,f). These results demonstrate that the native promoter, driving expression at physiologically appropriate levels with proper spatiotemporal regulation, represents the optimal choice. Constitutive strong promoters such as *CaMV35S*, while enhancing signal intensity for easier screening, carry inherent risks of artifacts and should be used with caution.

**Figure 3:**
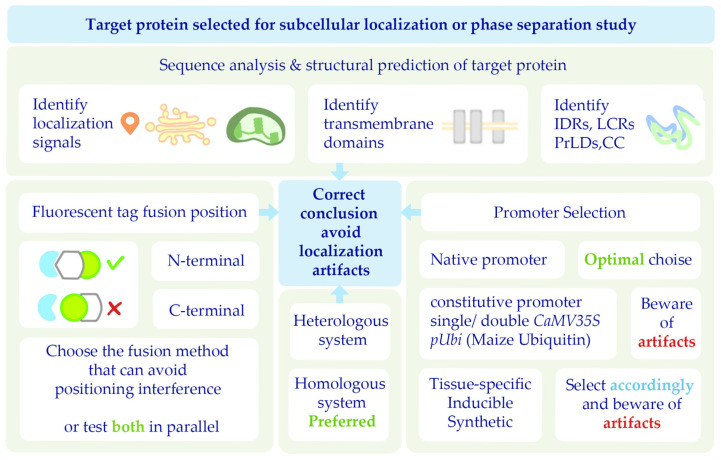
Guideline for protoplast transformation system selection. The schematic diagram of the fluorescent tag fusion position suggests that the fluorescent protein tag does not block recognition of the localization signal.

Through integrating previous reviews, conducting systematic evaluations, and synthesizing empirical validation, we established a practical guideline for protein subcellular localization studies in wheat protoplasts (Figure 3). The workflow begins with sequence analysis and structural prediction of the target protein to identify localization signals, transmembrane domains, and intrinsically disordered regions (IDRs). Based on this information, the appropriate fluorescent tag fusion position (N- or C-terminus) should be selected to avoid obstructing critical targeting signals; improper tag placement may sterically interfere with localization signals [39], disrupt weak multivalent interactions mediated by IDRs and their conformational flexibility [40,41], or affect post-translational modification and protein stability [42]. When uncertain, parallel testing of both configurations is recommended. Promoter choice represents an equally critical decision point: the native promoter is the optimal choice for physiological accuracy, as phase separation behavior is highly concentration-dependent [43,44]. Overexpression driven by strong promoters can elevate protein levels beyond the physiological range, exacerbate molecular crowding, and induce non-physiological puncta that may be mistaken for authentic condensates [45]. Ideally, the native promoter best reflects true localization, but in transient transformation the signal is often too weak. Strong promoters such as 35S are more practical, but must be used with caution and appropriate controls, balancing signal intensity against physiological accuracy. Constitutive promoters such as single/double *CaMV35S* or *pUbi*, as well as tissue-specific, inducible, and synthetic promoters, should be selected according to experimental needs and used with caution against artifacts. Furthermore, the homologous wheat protoplast system is preferred over heterologous systems, which may introduce localization artifacts due to divergent intracellular environments. Finally, validation by immunofluorescence or other endogenous detection methods is recommended as a calibration step to ensure that the conclusions reflect the physiological reality.

## 4. Materials and Methods

### 4.1. Plant Material

Wheat etiolated seedlings (dark-cultured for 8–12 days, plant height 15–25 cm, healthy growth, pest-free, and chlorophyll-free; aerial parts are used for protoplast preparation).

### 4.2. Chemical and Stock Solutions

Storage notes: Mannitol can be sealed and stored at room temperature due to easy crystallization at 4 °C; other stock solutions could be stored at 4 °C; PEG-Ca^2+^ transfection solution stored at 4 °C and used within one month; W5 buffer stored at room temperature; and enzymatic hydrolysis solution freshly prepared before use.

### 4.3. Experimental Instruments

Centrifuge (equipped with horizontal rotor), adjustable volume pipettes (10 μL to 1 mL) and tips, sterile Petri dishes (60 mm), 50 mL centrifuge tubes, 2 mL centrifuge tubes, 75 μm nylon mesh filter, constant temperature shaker, Nikon Ni-U upright fluorescence microscope, Nikon A1 HD25/A1R HD25 confocal microscope, vacuum desiccator, constant temperature water bath, magnetic stirrer, and 0.45 μm aqueous filter.

### 4.4. Transformation Vectors

Confirmation that the protoplast transformation system is functional: pCambia1305-2 × CaMV35S-eGFP, pCambia1300-2 × CaMV35S-PIF-mCherry, pCambia1300-2 × CaMV35S-HDEL-mCherry, pCambia1300-2 × CaMV35S-CBL-mCherry.

Validation of transformation effect: pCambia1305-2 × CaMV35S-eGFP.

Validation of tag position effect on protein localization: pJIT163-2 × CaMV35S-eGFP-TaPLGG1 and pCambia1305-2 × CaMV35S-TaPLGG1-eGFP.

Validation of protein expression level effect on protein localization: pCambia1305-2 × CaMV35S-TaSUVR2-eGFP and pCambia1305-CaMV35S-TaSUVR2-eGFP.

### 4.5. Plant Material Preparation

Wheat seeds are dehulled, dried, and planted in pots. This method is not limited by variety; common wheat varieties such as Chinese Spring, Fielder, ALCD, and their transgenic lines’ materials can be used.

Place the pots in a dark incubator and culture at room temperature for 8–12 days to obtain etiolated seedlings.

**Figure 4:**
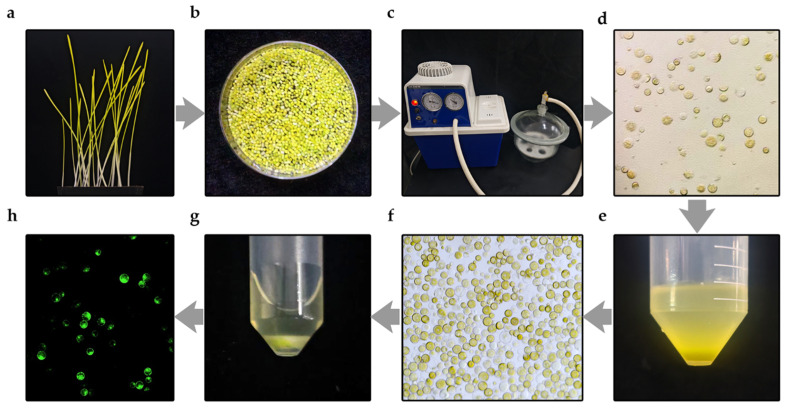
Protoplast isolation and PEG-mediated transformation in wheat. (**a**) Wheat seedlings grown in darkness for 8–12 days. (**b**) Wheat leaves cut into strips and floated in enzyme solution. (**c**) Vacuum infiltration treatment. (**d**) Unconcentrated protoplast suspension after enzymatic digestion. (**e**) Protoplasts in W5 solution after settling on ice (the yellowish-green pellet at the bottom indicates high-quality protoplasts). (**f**) Protoplasts resuspended in MMG solution. (**g**) PEG–Ca^2+^-mediated transformation. (**h**) Free GFP expression in transformed protoplasts.

Select healthy etiolated seedlings with a plant height of 15–25 cm, cut the aerial parts and remove the tips for protoplast preparation (Figure 4a).

### 4.6. Protoplast Isolation

1. Prepare the stock solution before lysing the protoplasts (Table 1), and prepare it fresh for immediate use. Minimize light exposure throughout the process and take care to reduce protoplast damage.

2. Use a scalpel blade to cut the aerial parts of wheat etiolated seedlings into 1 mm-wide strips, and immediately place them into a 35 mm sterile Petri dish containing 10 mL enzymatic hydrolysis solution (Table 2), ensuring the plant tissue is completely submerged (Figure 4b). Image shows reference size of leaf strips.

3. Wrap the Petri dish with aluminum foil, place it in a vacuum desiccator, and apply a vacuum below −0.85 MPa for 25 min (Figure 4c).

4. Slowly release the vacuum, then place the Petri dish on a shaker and digest in the dark at room temperature, 40 rpm for 2–4 h. Start microscopic observation 2 h after digestion begins. When densely distributed protoplasts are visible in the microscope field (Figure 4d), the image shows a reference for termination of digestion; increase the shaker speed to 100 rpm for 10 min to fully release the protoplasts.

5. Add an equal volume (10 mL) of ice-cold W5 buffer (Table 2) to the Petri dish, and gently mix by inverting to stop the enzymatic reaction.

6. Filter the enzymatic digest through a 75 μm nylon mesh filter into a 50 mL sterile centrifuge tube to remove undigested tissue fragments.

7. Centrifuge at 100× *g* for 5 min at 4 °C, with the centrifuge acceleration and deceleration set to the lowest settings, to pellet the protoplasts. Carefully aspirate the supernatant, leaving about 1 mL of liquid, avoiding aspiration of the protoplast pellet.

8. Add 10 mL of ice-cold W5 buffer, gently resuspend the protoplast pellet by inverting, and let it sit on ice for 30 min. After natural sedimentation of protoplasts, carefully aspirate the supernatant again to remove as many impurities as possible (this natural sedimentation step helps obtain high-quality protoplasts) (Figure 4e).

9. Add 1–2 mL MMG solution (Table 2), gently resuspend the pellet by inverting. Observe protoplast density under a phase-contrast microscope and adjust the amount of MMG solution accordingly (Figure 4f); the image shows a reference for the resuspension density.

### 4.7. Protoplast Transformation

1. Take a 2 mL centrifuge tube and add 30–40 μg of plasmid DNA (endotoxin must be removed during maxi-preparation) (concentration 2000 ng/μL, volume 10–20 μL).

2. Use a cut tip to add 200 μL of protoplast suspension and gently mix by inverting to ensure full contact between the protoplasts and DNA.

3. Add an equal volume (210–220 μL) of PEG-Ca^2+^ solution (Table 2) to the total volume of plasmid and protoplasts, and gently mix by inverting.

4. Incubate at room temperature (22–25 °C) in the dark for 16 min without shaking (Figure 4g).

5. Slowly add 1000 μL of ice-cold W5 buffer and gently mix by inverting to stop the transformation reaction.

6. Centrifuge at 100× *g* for 3 min at 4 °C (set centrifuge acceleration and deceleration to 1); collect protoplasts. Carefully aspirate the supernatant, leaving a small amount of liquid to resuspend the pellet.

7. Add 1 mL W5 buffer and gently resuspend the protoplast pellet by inverting.

8. Wrap the centrifuge tube containing the protoplast suspension with aluminum foil and incubate at room temperature for 16–20 h.

9. After incubation, centrifuge at 100× *g* for 3 min and carefully aspirate about 800 μL of the supernatant.

10. Use a cut tip to gently pipette the protoplast pellet at the bottom of the tube to resuspend. Take a small amount of the resuspension (about 20 μL) and drop onto a glass slide. No fixation is needed; proceed directly to subsequent observation (Figure 4h). Counting from three independent replicates showed that the transformation efficiency of wheat protoplasts using the method described herein is approximately 30% to 45%. Following the recommended mounting procedure, no fewer than 40 successfully transformed protoplasts can be observed per field of view at 100× magnification.

### 4.8. Experimental Precautions

1. Etiolated seedlings must be cultured in complete darkness to avoid cell wall lignification and chlorophyll synthesis, reducing interference from chloroplast autofluorescence during fluorescent protein observation. Also, select healthy, vigorously growing seedlings that are free from pests and diseases.

2. The composition of the enzymatic hydrolysis solution, digestion temperature, and time are key factors affecting protoplast isolation efficiency. Over-digestion causes protoplast rupture, while under-digestion results in incomplete cell digestion. Therefore, adjust the digestion time based on the material state and enzyme activity.

3. Protoplasts are fragile. Therefore, use lower centrifugal speed (typically 100× *g*) and shorter time (typically 3 min) to avoid rupture. Also, use lower acceleration and deceleration rates (adjustable to 1) to allow protoplasts to settle at the bottom of the tube, preventing loss when removing the supernatant after centrifugation due to weak adherence. Handle liquids containing protoplasts with great care, using cut tips to avoid shear force that may cause protoplast breakage.

4. Plasmids used for protoplast transformation must be endotoxin-free during maxi-preparation. After extraction, check the plasmid concentration and purity, ensuring that the OD260/OD280 ratio is between 1.8 and 2.0 and the concentration is no less than 2000 ng/μL. Store extracted plasmids at −20 °C and avoid repeated freeze–thaw cycles. Thaw at room temperature and briefly centrifuge to collect the liquid before use.

5. Use 40% PEG-4000 and control incubation time to 16 min. Higher concentrations or longer incubation times increase protoplast toxicity, reducing the yield of high-quality protoplasts.

6. Strict sterile operation is not required throughout the experiment, but care should be taken to prevent excessive microbial growth. When room temperature is high in summer, the working concentration of antibiotics can be added to W5 to inhibit microbial proliferation.

7. Laser confocal microscopes are often inverted microscopes, so the suspension can be dropped onto a coverslip with better transparency, and carefully cover it with another coverslip. Once the coverslip is applied, its position cannot be adjusted; otherwise, it may cause protoplast breakage.

### 4.9. Transformation Efficiency Detection and Live-Cell Image Analysis

Live protoplasts were imaged using a laser confocal microscope 16–20 h after transformation. All samples are uniformly analyzed for expression of the reporter gene (*GFP*) to assess protoplast transformation efficiency and observation and recording of fluorescence signal intensity and distribution patterns to determine the subcellular localization characteristics of the target protein. To validate the physiological relevance of the phenomenon observed in the overexpression system, one gene exhibiting a clear and reproducible punctate aggregation phenomenon in the overexpression system was selected. Immunofluorescence staining was performed on non-transformed protoplasts subjected to the same treatment, using antibody detection results as a preliminary control.

## 5. Conclusions

This study established a simplified wheat protoplast transformation system with a short experimental duration and high protoplast quality, suitable for protein subcellular localization and phase separation analyses. When constructing fusion expression vectors, correct fluorescent tag fusion is essential for recapitulating endogenous localization patterns, and promoter selection also requires careful consideration—the native promoter provides the most reliable reference for physiological distribution and condensate formation. The vector design guidelines and standardized system defined in this study constitute an experimental framework that minimizes artifacts and enhances reliability, which is applicable to protein localization and phase separation research in wheat and other recalcitrant plant species.

## Figures and Tables

**Table 1:** Stock solution.

Component	Stock Solution	Volume	Preparation
0.2 M MES (pH 5.7)	1.25 M MES (pH 5.7)	50 mL	1.95 g MES (Cat. No. M5287, Sigma-Aldrich, China) powder dissolved in ultrapure water; pH adjusted to 5.7 with KOH; volume made up to 50 mL
0.8 M Mannitol	–	50 mL	7.29 g mannitol (Cat. No. 63560-250G-F, Sigma-Aldrich) dissolved in ultrapure water; volume made up to 50 mL
1 M CaCl_2_	Anhydrous CaCl_2_	50 mL	5.55 g anhydrous CaCl_2_ (Cat. No. C5670-100G, Sigma-Aldrich, USA) dissolved in ultrapure water; volume made up to 50 mL
2.5 M NaCl	–	20 mL	2.92 g NaCl (Cat. No. S3014-500G, Sigma-Aldrich, China) dissolved in ultrapure water; volume made up to 20 mL
2 M KCl	–	20 mL	2.98 g KCl (Cat. No. P5405-250G, Sigma-Aldrich, China) dissolved in ultrapure water; volume made up to 20 mL
2 M MgCl_2_	Anhydrous MgCl_2_	20 mL	3.8 g anhydrous MgCl_2_ dissolved in ultrapure water; volume made up to 20 mL

**Table 2:** Working solution.

Buffer	Volume	Preparation Method
Enzymatic hydrolysis solution	20 mL	2 mL 0.2 M MES (pH 5.7, preheated at 70 °C for 3–5 min) + 0.3 g Cellulase Onozuka R-10 (Yakult Pharmaceutical, Japan) (1.5% *m*/*v*) + 0.08 g Macerozyme R-10 (Yakult Pharmaceutical, Japan) (0.4% *m*/*v*) + 10 mL 0.8 M Mannitol + 200 μL 2 M KCl; incubated at 55 °C for 10 min; cooled to room temperature; 200 μL 1 M CaCl_2_ + 0.02 g BSA (Cat. No. B2064-100G, Sigma-Aldrich, New Zealand) (0.1% *m*/*v*) added; filtered through a 0.45 μm filter before using.
W5 buffer	100 mL	1 mL 0.2 M MES (pH 5.7) + 6.16 mL 2.5 M NaCl + 12.5 mL 1 M CaCl_2_ + 250 μL 2 M KCl; volume made up to 100 mL with ultrapure water.
PEG-Ca^2+^ transfection solution	10 mL	2.5 mL 0.8 M Mannitol + 1 mL 1 M CaCl_2_ + 3 mL ultrapure water + 4 g PEG-4000 (Cat. No. 95904-250G-F, Sigma-Aldrich, Switzerland) (40% *m*/*v*); magnetic stirred for 1–2 h to dissolve completely; volume made up to 10 mL.
MMG solution	20 mL	400 μL 0.2 M MES (pH 5.7) + 10 mL 0.8 M Mannitol + 150 μL 2 M MgCl_2_; volume made up to 20 mL with ultrapure water.

## Data Availability

The data and methods presented in this study are included in the Materials and Methods section. Further inquiries can be directed to the corresponding author.

## Abbreviations

The following abbreviations are used in this manuscript:

Abbreviation	Full Name
BSA	Bovine Serum Albumin
*CaMV35S*	Cauliflower Mosaic Virus 35S Promoter
eGFP	Enhanced Green Fluorescent Protein
MES	2-(N-Morpholino) ethanesulfonic Acid
MMG	MES-Mannitol-MgCl_2_ Buffer
PEG	Polyethylene Glycol
W5	Washing Buffer 5
